# Accuracy of lung ultrasound performed with handheld ultrasound device in internal medicine: an observational study

**DOI:** 10.1007/s40477-024-00941-5

**Published:** 2024-08-03

**Authors:** Anna Lo Cricchio, Andrea Storelli, Iacopo Bertoletti, Gabriele Ciuti, Alessia Fabbri, Elisa Martinelli, Maria Cristina De Santis, Paolo Mercatelli, Khadija El Aoufy, Silvia Bellando Randone, Alberto Moggi Pignone, Esterita Accogli, Giulia Bandini

**Affiliations:** 1 Department of Experimental and Clinical Medicine, University of Florence, Careggi Hospital, Florence, Italy; 2 Department of Health Science, University of Florence, Florence, Italy; 3 Department of Experimental and Clinical Medicine, Division of Rheumatology, University of Florence, Florence, Italy; 4 Department of Internal Medicine, Centre of Research and Learning in Ultrasound, Maggiore Hospital, Bologna, Italy

**Keywords:** Lung ultrasound, Point of care ultrasound, Handheld ultrasound device, Pocket-sized ultrasound, Heart failure, pneumonia

## Abstract

**Aims:**

Lung ultrasound (LUS) is increasingly used in Internal Medicine to complement medical examination, documenting pleural and lung conditions. This study aimed to compare the accuracy of handheld ultrasound device (HHUSD) with high-end ultrasound device (HEUSD) in patients with heart failure or pneumonia, also including the assessment of costs and time-savings.

**Methods:**

In this observational study 72 patients (aged ≥ 18) admitted to Internal Medicine Unit for heart failure or pneumonia underwent LUS plus evaluation of inferior cava vein (ICV) when indicated, using both HHUSD and HEUSD. Each evaluation, independently performed by 2 different experienced operators, included B-lines number, pleural effusion, lung consolidations, ICV ectasia and its respiratory excursions.

**Results:**

Concordance between HHUSD and HEUSD findings was 79.3% ± 17.7 (mean ± SD) for B-lines, 88.6% for pleural effusion, 82.3% for consolidations and 88.7% and 84.9% for ICV ectasia and its respiratory excursions respectively. BMI didn’t significantly influence concordance between the two methods. Moreover, examination time (as mean ± SD) was shorter with HHUSD (8 ± 1.5 min) compared to HEUSD (10 ± 2.5 min).

**Conclusions:**

HHUSD demonstrated high accuracy in detecting B-lines, pleural effusions, lung consolidations and ICV evaluation when compared to HEUSD. Thus, HHUSD, not only is characterized by accessibility, portability, and easy handling due to its small size, but it also offers advantages in terms of saving costs and time, ultimately contributing to faster patient assessment compared to HEUSD.

## Introduction

In recent years, lung ultrasound (LUS) has emerged as a reliable and rapid tool for the evaluation of patients with pulmonary diseases [1]. LUS, plus the inferior cava vein (ICV) assessment, can improve the diagnosis of many cardiopulmonary conditions such as pleural effusion, interstitial lung disease and pneumonia; moreover, LUS can guide procedures (i.e. thoracentesis), drive therapeutic timing and dosage (i.e. diuretic therapy) and it is a valid instrument for monitoring and prognosis of patients with heart failure [2–5].

In the last few years, the development and spread of knowledge in the field of LUS and the expansion of inexpensive and handy tablet or smartphone/tablet format devices, have made the point-of-care ultrasound (POCUS) approach become a cornerstone in the evaluation of patients with respiratory symptoms. Nowadays, LUS is a fundamental supplement to the medical examination. Indeed, pocket-sized devices are frequently used in the bedside evaluation of hospitalized patients, however, their use is rapidly increasing even in ambulatory settings to answer simple clinical questions especially thanks to their low cost and good performance profiles [6–8].

These days, ultrasound is considered an essential aspect of bedside examination since it can help to rapidly frame the patient [9] and accelerate the diagnostic therapeutic pathway.

However, despite the widespread of poket-sized devices thanks to new technologies, with a major boost for their development during the SARS-CoV2 pandemic, to date there are no solid data to support the interchangeable use between high-end ultrasound devices (HEUSDs) and handheld ultrasound devices (HHUSDs), except for few papers comparing the two methods [10–12].

Despite technological advancements enabling the development of increasingly efficient pocket-sized ultrasound machines, the literature still reports lower performance of these devices in terms of image quality compared to HEUSDs. This discrepancy is attributed to various factors, including lower spatial resolution, reduced contrast and higher levels of noise. Specifically, the literature highlights a reduced penetration depth of the ultrasound beams produced by these devices [13, 14].

Thus, the aim of our work was to evaluate the accuracy of LUS performed with HHUSD compared to HEUSD in patients admitted to our ward for heart failure or pneumonia and to determine if the pocket-sized ultrasound approach has advantages in terms of saving costs and time. Then, we considered whether obesity may be a limiting condition for HHUSDs due to increased fat layer and reduced penetration depth of the sound beams of these machines.

## Materials and methods

We conducted in a single center an observational study involving adults hospitalized in the Department of Internal Medicine 4 of the Careggi University Hospital in Florence.

Over 6 months, 72 patients were enrolled. For each patient demographic, clinical and laboratory data were recorded.

The enrolled patients underwent LUS plus the evaluation of ICV, when indicated, both performed with the HHUSD Vscan Extend Dual probe (GE Healthcare) with a phased array transducer (1.7–3.8 MHz) and a linear (3.3–8 MHz) transducer. and the HEUSD Vivid T8 (GE Healthcare) with a convex transducer (3.5–7 MHz). Ultrasound evaluations were performed independently by two different operators experienced in LUS, at closely spaced times (a maximum of 15 min apart) using a standardized imaging protocol [15].

Every patient was scanned in the supine and sitting position and, for each of the 58 areas examined, the following data were registered: number of B-lines, quantified as suggested by the literature [16, 17], the total number of B-lines resulting from the evaluation of each of the spaces explored for the antero-lateral chest and for the posterior chest, the presence of pleural effusion and/or lung consolidation. Moreover, in patients with heart failure, inferior cava vein ectasia (diameter > 20 mm) and its respiratory excursions, defined as maintained if greater than 50% of the diameter, were recorded.

Finally, the duration of each examination was registered.

The data obtained by the two types of ultrasounds were then compared, and it was assessed whether there was overlap between the findings identified by the two different methods. The number of B-lines for each field explored was judged to be overlapping if there was a numerical difference ≤ 2.

### Statistical analysis

Continuous normal variables have been expressed as mean and standard deviation (SD), and non-normal variables as median (minimum value-maximum value). Categorical variables have been expressed as number and percentage. Comparison between groups have been performed with the chi-square test for dichotomous variables. Wilcoxon test have been performed for paired continuous variables for comparison of HEUSD and HHUSD. A *p*-value < 0.05 have been considered statistically significant. All statistical analyses have been performed using SPSS software version 20.0 (IBM, Armonk, New York, USA).

## Results

**Fig. 1:**
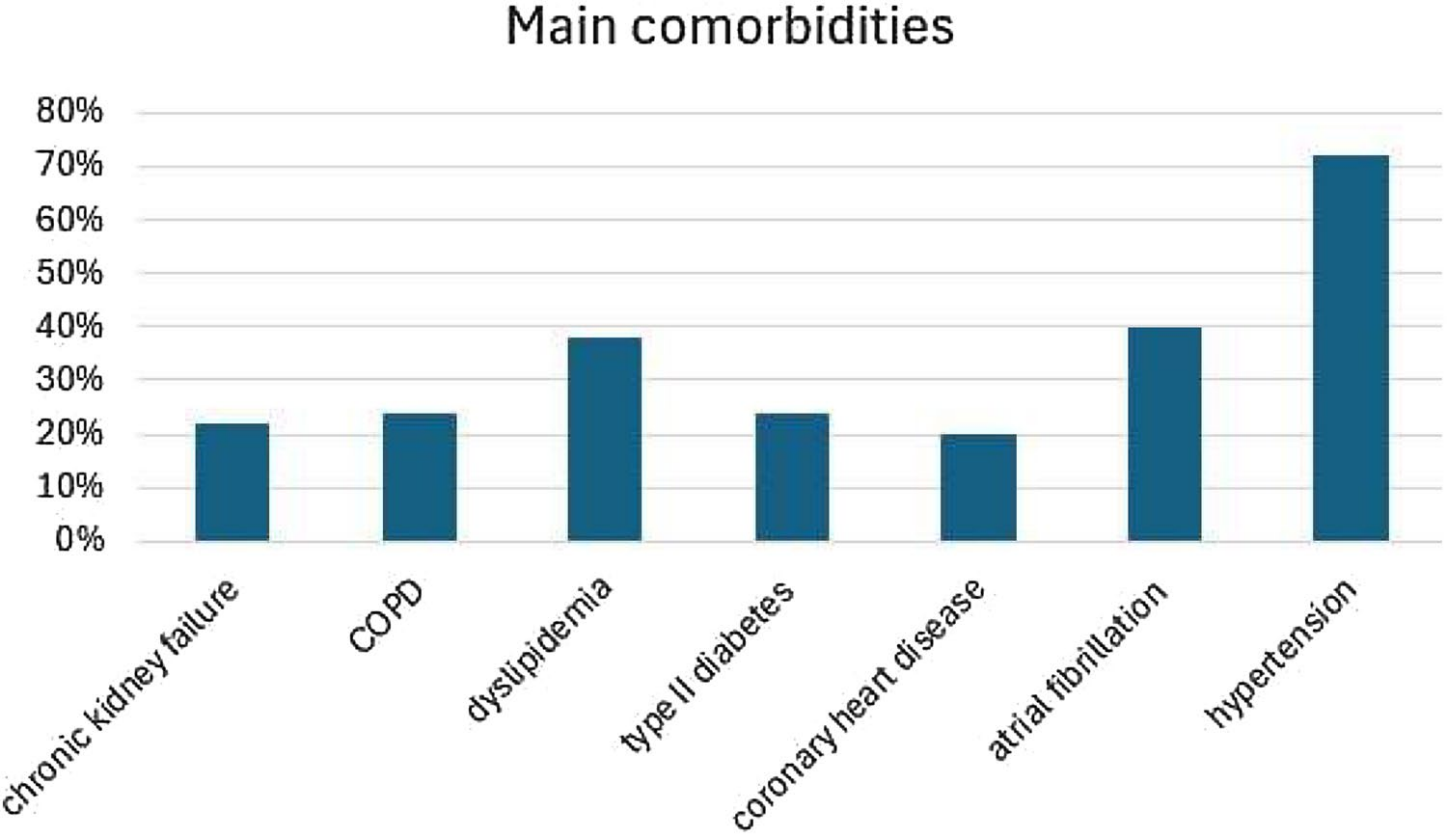
Main comorbidities of enrolled patients

Seventy-two patients with a median age of 80 years (range 28–99), among which 39 males (54%), were enrolled in the study. Admission diagnosis to our ward were heart failure (68%) or pneumonia (32%). The principal comorbidities were hypertension (72%), atrial fibrillation (40%), type II diabetes (24%), dyslipidemia (38%), chronic obstructive pulmonary disease (24%), chronic kidney failure (22%) and coronary heart disease (20%) (Fig. 1).

94% of patients had at least one of the following cardiovascular risk factors: obesity, smoking, hypertension, diabetes, dyslipidemia.

The most frequently prescribed medications upon admission to the ward were: diuretics (60%), beta-blockers (54%), calcium-antagonists (28%), direct anticoagulants (28%), angiotensin converting enzyme (ACE) inhibitors (20%), sartans (16%), and warfarin (4%).

In 70% of the cases, physical examination revealed the presence of wet sounds upon chest auscultation, while in the remaining 30% bronchial obstruction sounds; moreover, 55% of patients had swollen limbs or feet.

The main laboratory alterations upon admission to the ward were an increase in NT-proBNP, troponin, and C-reactive protein (Table 1).

**Table 1:** Laboratory tests of enrolled patients available at the admission ( ↑ increased values, ↓ decreased values)

	Patients analyzed * n* (%)	Patients with abnormal values * n* (%)
NT-proBNP (n.v. 1–125 pg/mL)	57 (79.2%)	↑ 52 (97.1%)
Creatinine (n.v. 0.70–1.20 mg/dL)	72 (100%)	↑ 25 (34.7%)
Troponin T (n.v. < 14 pg/mL)	50 (69.4%)	↑ 43 (86%)
CRP (n.v. < 5 mg/L)	67 (93.1%)	↑ 62 (92.5%)
PCT (n.v. < 0.5 ng/mL)	65 (90.3%)	↑ 6 (9.2%)
Hb (n.v. 14.0–18.0 g/dL)	72 (100%)	↓ 59 (82%)
WBC (n.v. 4.00–10.00 × 10^9^/L)	72 (100%)	↑ 20 (28%)
Hypokaliemia (K^+^ < 3.5 mEq/L)	72 (100%)	9 (12.5%)
Hyperkaliemia (K^+^ > 5.1 mEq/L)	72 (100%)	2 (2.8%)
Hyponatriemia (Na^+^ < 135 mEq/L)	72 (100%)	12 (16.7%)
Hypernatriemia (Na^+^ > 145 mEq/L)	72 (100%)	6 (8.3%)
AST (n.v. 10–50 U/L)	44 (61.1%)	↑ 10 (22.7%)
ALT (n.v. 10–50 U/L)	70 (97.2%)	↑ 9 (12.9%)

*NT-proBNP*  N-terminal prohormone of brain natriuretic peptide, *CRP* C-reactive protein, *PCT* procalcitonin, *Hb* hemoglobin, *WBC* white blood cells, *K*^*+*^  potassium, *Na*^*+*^  sodium, *AST* aspartate transaminase, *ALT* alanine transaminase, *n.v.* normal value

The comparison between the HHUS and the HEUS evaluation showed a concordance rate of 79.3% ± 17.7 (mean ± SD) for the detection of B-lines, 88.6% for pleural effusion and 82.3% for lung consolidations.

Concordance rate between the two methods in the evaluation of ICV ectasia and its respiratory excursions were 88.7% and 84.9%, respectively.

BMI was available for 69 out of the 72 patients, in which 20% had BMI > 30 kg/m^2^. In this subgroup of patients, the concordance rate between the 2 methods was 78.9% ± 12.6 for the detection of B-lines, 86.5% for pleural effusion, 79.5% for consolidations, 86.3% and 85.8% for the evaluation of ICV ectasia and its respiratory excursions respectively. Between the two groups (patients with BMI > 30 kg/m^2^ and patients with BMI < 30 kg/m^2^), there were no statistically significant differences (*p* = 0,643) in LUS and ICV evaluation.

Data about concordance rates between the HHUSD and the HEUSD evaluation are reported in Table 2.

**Table 2:** Concordance rate between handheld ultrasound device (HHUSD) and high-end ultrasound device (HEUSD) in B-lines detection was expressed in mean ± standard deviation. Concordance rates between the two devices for the dection of pleural effusion and lung consolidations and for inferior cava vein (ICV) assessment were expressed as number and their percentages

Ultrasound findings detected	Concordance between HHUSD and HEUSD	
	Total enrolled population	Patients with BMI > 30 kg/m^2^
B-lines	79.3% ± 17.7	78.9% ± 12.6
Pleural effusion	88.6%	86.5%
Lung consolidations	82.3%	79.5%
Presence/absence of ICV ectasia	88.7%	86.3%
Respiratory excursions of the ICV (> or < 50% of the diameter)	84.9%	85.8%

The average time taken to perform the evaluations (expressed as mean ± SD) was 8 ± 1.5 min with the HHUSD and 10 ± 2.5 min with the HEUSD, with a statistically significant difference (*p* < 0.0001).

## Discussion

Bedside ultrasound has become a fundamental diagnostic tool in Internal Medicine care setting, and it has profoundly changed the clinical practice and the approach to patients’ evaluation and management. In particular, LUS is an essential part of POCUS which is increasingly considered as an extension of the physical examination, leading to the modern concept of ultrasound-assisted patients examination [9]. POCUS is, indeed, able to acceleratethe diagnostic and therapeutic process and to guide the management of inpatients in Internal Medicine settings.

In recent years, pocket-sized ultrasound devices are increasingly used in many different clinical settings given their low cost and technological evolution. HHUSDs present several advantages such as their low costs and rapid use, their feasibility and the possibility of always having them at hand due to their small size, that have contributed to their widespread use in numerous hospital and non-hospital settings [10, 18]. Moreover, the use of a pocket-sized, lightweight, and handy device in everyday clinical practice can provide the operator with greater ergonomics due to the smaller spatial footprint, and this may contribute to making healthcare professionals’ movements more comfortable [19, 20].

The use of a pocket-sized device, in daily clinical-assistance activities, can then help reducing physical stress for sonographers by reducing repetitive strain injuries that, to date, are becoming a perceived, and sometimes, disabling issue for physicians performing numerous ultrasound evaluations due to having non-ergonomic positions in environments where space is often limited.

However, the performance of HHUSDs in everyday clinical practice and their role in the evaluation of patients with heart failure and pneumonia hasn’t still been clearly defined.

**Fig. 2:**
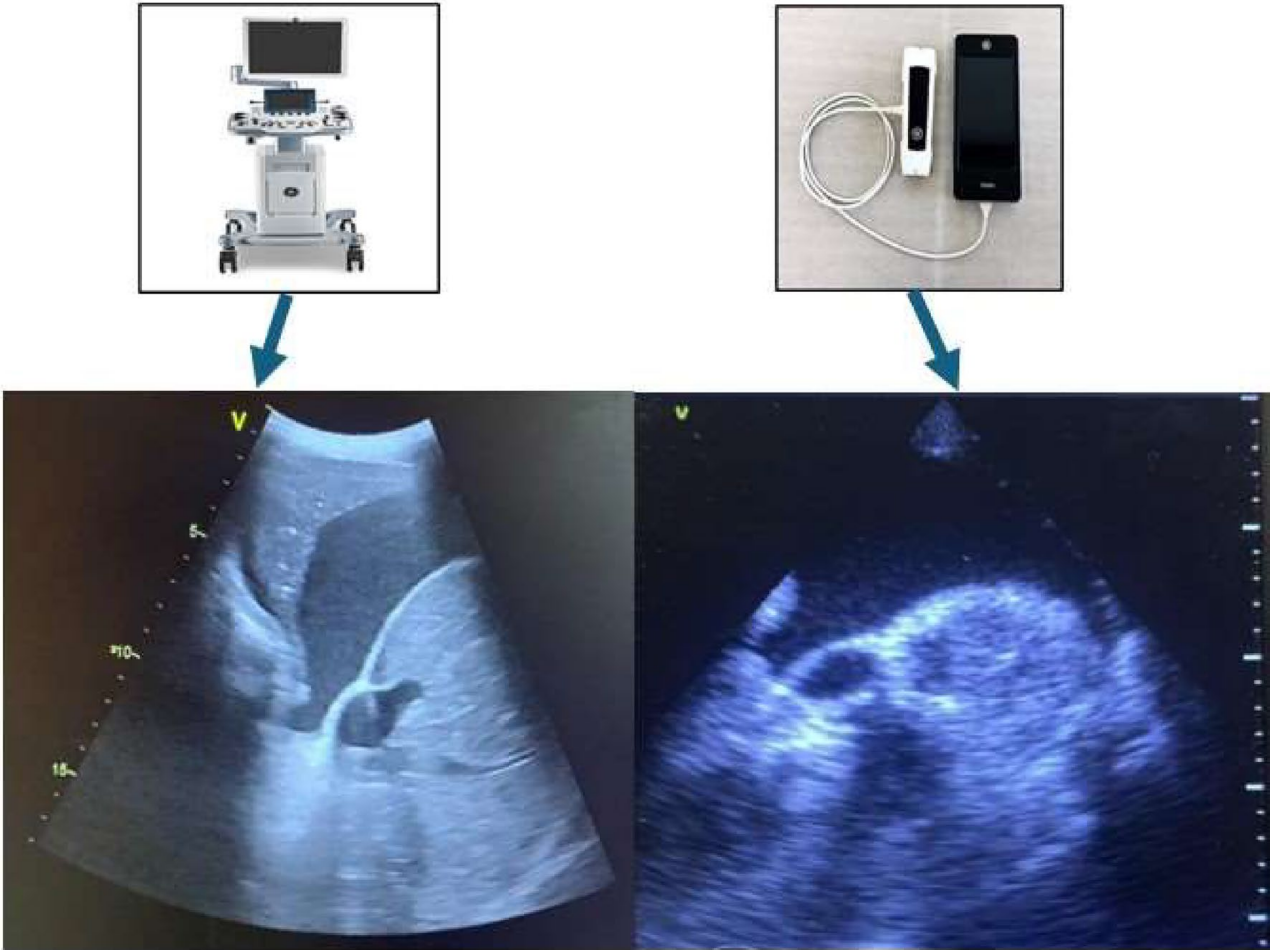
On the left, a pleural effusion detected using the high-end ultrasound machine; on the right, a pleural effusion highlighted with the handheld ultrasound device

Our data show that the accuracy of LUS performed with HHUSD is high when compared with HEUSD in the evaluation of patients with heart failure/lung disease. In particular, pocket-sized device confirms its accuracy in the evaluation of B-lines and pleural effusion [21] (Fig. 2). Moreover, in our study even the evaluation of ICV ectasia and its respiratory excursions is accurate with HHUSD compared to HEUSD. ICV evaluation, together with LUS, is fundamental in the management of patients with heart failure for diagnosis, monitoring and even during the follow up and to adjust diuretic therapy. To the best of our knowledge, this is the first study in which both LUS and ICV findings with pocket-sized ultrasound device are evaluated in comparison with a HEUSD. The substantial overlap, in the reported data, highlights the validity of the assessments performed with the pocket-sized ultrasound devices, thus confirming the reliability of this technique in the management of patients with heart failure. Moreover, there was a substantial correspondence even in the evaluation of lung consolidations; indeed, despite the limits of LUS for the assessment of consolidations [22], HHUSD can be a valid option for the management of patients with pneumonia.

In addition, the average time to perform the examination with the pocket-sized device is less than the time taken with the high-end machine, which can contribute to faster and more efficient ultrasound assessment at the patient’s bedside.

Finally, our study shows that the accuracy of pocket-sized ultrasound devices is still relevant in the evaluation of patients with higher BMIs, making these portable instruments reliable also in this subgroup of patients.

Our findings support the interchangeable use of HHUSD and HEUSD in the management of inpatients with heart failure and pneumonia.

Our study has some limitations; indeed, all the ultrasound examinations were performed by expert lung ultrasound operators, and this may have contributed to increasing the accuracy of ultrasound examinations performed with the HHUSD. Then, our study has been conducted in a single center and the sample size is small, thus further studies will be needed to confirm the interchangeability of the two methods in larger populations.

## Conclusions

In conclusion, this study shows that LUS, performed with a pocket-sized ultrasound device has high accuracy with regard to the detection of B-lines, pleural effusion and lung consolidations. Moreover, it allows adequate assessments of inferior cava vein. The accuracy remains high also in obese patients. Handheld ultrasound devices can be confidently used for the management of inpatients with heart failure and/or pneumonia. Given the usefulness of these tools, specific operators’ training should be developed and encouraged to spread the HHUS devices use in clinical practice.

## Data Availability

All datasets generated or analysed during the current study are available from the corresponding author on reasonable request.

## References

[1] Laursen CB, Clive A, Hallifax R, Pietersen PI, Asciak R, Davidsen JR et al (2021) European respiratory society statement on thoracic ultrasound. Eur Respir J 57:2001519. 10.1183/13993003.01519-2020 33033148 10.1183/13993003.01519-2020

[2] Soni NJ, Franco R, Velez MI, Schnobrich D, Dancel R, Restrepo MI et al (2015) Ultrasound in the diagnosis & management of pleural effusions. J Hosp Med 10:811–816. 10.1002/jhm.2434 26218493 10.1002/jhm.2434 PMC4715558

[3] Gargani L (2019) Ultrasound of the lungs: more than a room with a view. Heart Fail Clin 15:297–303. 10.1016/j.hfc.2018.12.010 30832819 10.1016/j.hfc.2018.12.010

[4] Pugliese NR, Mazzola M, Bandini G, Barbieri G, Spinelli S, De Biase N et al (2023) Prognostic role of sonographic decongestion in patients with acute heart failure with reduced and preserved ejection fraction: a multicentre study. J Clin Med 12:773. 10.3390/jcm12030773 36769421 10.3390/jcm12030773 PMC9917462

[5] Kajimoto K, Madeen K, Nakayama T, Tsudo H, Kuroda T, Abe T (2012) Rapid evaluation by lung-cardiac-inferior vena cava (LCI) integrated ultrasound for differentiating heart failure from pulmonary disease as the cause of acute dyspnea in the emergency setting. Cardiovasc Ultrasound 10:49. 10.1186/1476-7120-10-49 23210515 10.1186/1476-7120-10-49 PMC3527194

[6] Galderisi M, Santoro A, Versiero M, Lomoriello VS, Esposito R, Raia R et al (2010) Improved cardiovascular diagnostic accuracy by pocket size imaging device in non-cardiologic outpatients: the NaUSiCa (Naples Ultrasound Stethoscope in Cardiology) study. Cardiovasc Ultrasound 8:51. 10.1186/1476-7120-8-51 21110840 10.1186/1476-7120-8-51 PMC3003628

[7] Schleder S, Dendl L-M, Ernstberger A, Nerlich M, Hoffstetter P, Jung E-M et al (2013) Diagnostic value of a hand-carried ultrasound device for free intra-abdominal fluid and organ lacerations in major trauma patients. Emerg Med J 30:e20–e20. 10.1136/emermed-2012-201258 22518057 10.1136/emermed-2012-201258

[8] Razi R, Estrada JR, Doll J, Spencer KT (2011) Bedside hand-carried ultrasound by internal medicine residents versus traditional clinical assessment for the identification of systolic dysfunction in patients admitted with Decompensated Heart failure. J Am Soc Echocardiogr 24:1319–1324. 10.1016/j.echo.2011.07.013 21885245 10.1016/j.echo.2011.07.013

[9] Narula J, Chandrashekhar Y, Braunwald E (2018) Time to add a fifth pillar to bedside physical examination: inspection, palpation, percussion, auscultation, and insonation. JAMA Cardiol 3:346–350. 10.1001/jamacardio.2018.0001 29490335 10.1001/jamacardio.2018.0001

[10] Rykkje A, Carlsen JF, Nielsen MB (2019) Hand-held ultrasound devices compared with high-end ultrasound systems: a systematic review. Diagnostics (Basel) 9:61. 10.3390/diagnostics9020061 31208078 10.3390/diagnostics9020061 PMC6628329

[11] Haji-Hassan M, Lenghel LM, Bolboacă SD (2021) Hand-held ultrasound of the lung: a systematic review. Diagnostics (Basel) 11:1381. 10.3390/diagnostics11081381 34441315 10.3390/diagnostics11081381 PMC8392700

[12] Nielsen MB, Cantisani V, Sidhu PS, Badea R, Batko T, Carlsen J et al (2019) The use of handheld ultrasound devices - an EFSUMB position paper. Ultraschall Med 40:30–39. 10.1055/a-0783-2303 30577046 10.1055/a-0783-2303

[13] Wang R, Fang Z, Gu J, Guo Y, Zhou S, Wang Y et al (2019) High-resolution image reconstruction for portable ultrasound imaging devices. EURASIP J Adv Signal Process 2019:56. 10.1186/s13634-019-0649-x

[14] Fröhlich E, Beller K, Muller R, Herrmann M, Debove I, Klinger C et al (2020) Point of care ultrasound in geriatric patients: prospective evaluation of a portable handheld ultrasound device. Ultraschall Med 41:308–316. 10.1055/a-0889-8070 31026863 10.1055/a-0889-8070

[15] Gargani L, Romei C, Bruni C, Lepri G, El-Aoufy K, Orlandi M, et al (2022) Lung ultrasound B-lines in systemic sclerosis: cut-off values and methodological indications for interstitial lung disease screening. Rheumatology 61:SI56–64. 10.1093/rheumatology/keab801 10.1093/rheumatology/keab801 34698807

[16] Pellicori P, Platz E, Dauw J, ter Maaten JM, Martens P, Pivetta E et al (2021) Ultrasound imaging of congestion in heart failure: examinations beyond the heart. Eur J Heart Fail 23:703–712. 10.1002/ejhf.2032 33118672 10.1002/ejhf.2032 PMC8081753

[17] Volpicelli G, Elbarbary M, Blaivas M, Lichtenstein DA, Mathis G, Kirkpatrick AW et al (2012) International evidence-based recommendations for point-of-care lung ultrasound. Intensive Care Med 38:577–591. 10.1007/s00134-012-2513-4 22392031 10.1007/s00134-012-2513-4

[18] Ault MJ, Rosen BT (2010) Portable ultrasound: the next generation arrives. Crit Ultrasound J 2:39–42. 10.1007/s13089-010-0031-6 21151494 10.1007/s13089-010-0031-6 PMC2994643

[19] Coffin CT (2014) Work-related musculoskeletal disorders in sonographers: a review of causes and types of injury and best practices for reducing injury risk. Rep Med Imag 7:15–26. 10.2147/RMI.S34724

[20] Wilkinson JN, Saxhaug LM (2021) Handheld ultrasound in training - The future is getting smaller! J Intensive Care Soc 22:220–229. 10.1177/1751143720914216 34422105 10.1177/1751143720914216 PMC8373282

[21] Schleder S, Dornia C, Poschenrieder F, Dendl L, Cojocaru L, Bein T, Schmid C, Stroszczynski C, Jung EM, Heiss P (2012) Bedside diagnosis of pleural effusion with a latest generation hand-carried ultrasound device in intensive care patients. Acta Radiol 53(5):556–560. 10.1258/ar.2012.110676 22661602 10.1258/ar.2012.110676

[22] Reissig A, Görg C, Mathis G (2009) Transthoracic sonography in the diagnosis of pulmonary diseases: a systematic approach. Ultraschall Med 30:438–54; quiz 455–6. 10.1055/s-0028-1109703 10.1055/s-0028-1109703 19813156

